# Review of adaptive control for stroke lower limb exoskeleton rehabilitation robot based on motion intention recognition

**DOI:** 10.3389/fnbot.2023.1186175

**Published:** 2023-07-03

**Authors:** Dongnan Su, Zhigang Hu, Jipeng Wu, Peng Shang, Zhaohui Luo

**Affiliations:** ^1^Shenzhen Institutes of Advanced Technology, Chinese Academy of Sciences, Shenzhen, China; ^2^School of Medical Technology and Engineering, Henan University of Science and Technology, Luoyang, China; ^3^Henan Intelligent Rehabilitation Medical Robot Engineering Research Center, Henan University of Science and Technology, Luoyang, China; ^4^State-Owned Changhong Machinery Factory, Guilin, China

**Keywords:** stroke, lower limb exoskeleton, rehabilitation, motion intention recognition, adaptive control

## Abstract

Stroke is a significant cause of disability worldwide, and stroke survivors often experience severe motor impairments. Lower limb rehabilitation exoskeleton robots provide support and balance for stroke survivors and assist them in performing rehabilitation training tasks, which can effectively improve their quality of life during the later stages of stroke recovery. Lower limb rehabilitation exoskeleton robots have become a hot topic in rehabilitation therapy research. This review introduces traditional rehabilitation assessment methods, explores the possibility of lower limb exoskeleton robots combining sensors and electrophysiological signals to assess stroke survivors' rehabilitation objectively, summarizes standard human-robot coupling models of lower limb rehabilitation exoskeleton robots in recent years, and critically introduces adaptive control models based on motion intent recognition for lower limb exoskeleton robots. This provides new design ideas for the future combination of lower limb rehabilitation exoskeleton robots with rehabilitation assessment, motion assistance, rehabilitation treatment, and adaptive control, making the rehabilitation assessment process more objective and addressing the shortage of rehabilitation therapists to some extent. Finally, the article discusses the current limitations of adaptive control of lower limb rehabilitation exoskeleton robots for stroke survivors and proposes new research directions.

## 1. Introduction

Stroke is a major cause of disability and mortality globally (Feigin et al., 2022), often leading to motor impairments such as muscle spasms and movement disorders that can severely impact the quality of life of stroke survivors (Bansil et al., 2012). Rehabilitation therapy can aid in restoring motor function, improving mobility, and enhancing independence in performing daily activities (Wagenaar et al., 1990). However, to effectively design personalized rehabilitation plans and monitor progress, rehabilitation therapists need to assess the survivor's motor impairment at various stages (Wagenaar et al., 1990).

Lower limb rehabilitation exoskeleton robots are mechanical devices that can augment human movement or replace lost activity (Bogue, 2015). These robots have been designed to assist patients with impaired lower limb motor ability, including stroke survivors, in relearning to walk and restoring lower limb muscle strength (Shi D. et al., 2019). Exoskeleton robots for lower limb rehabilitation have become a research hotspot in the field of stroke rehabilitation due to their ability to support and maintain balance for stroke survivors while assisting them in completing rehabilitation training tasks (Li et al., 2015). However, to fully harness the benefits of lower limb rehabilitation exoskeleton robots, adaptive control algorithms that can adapt to different situations in survivors' daily lives or rehabilitation training are necessary (Bansil et al., 2012).

In recent years, there has been growing interest in using motion intention recognition algorithms to improve the adaptive control of lower limb exoskeleton robots in stroke rehabilitation (Masengo et al., 2023). These algorithms obtain stroke survivors' movement expectations through various Kinematic parameters or electrophysiological signals and adjust the exoskeleton robot's motion in real-time to provide motion intention (Liu et al., 2023). This adaptive control approach has shown promising results in improving the effectiveness of lower limb exoskeleton rehabilitation robots for stroke survivors (Li et al., 2020; Peng et al., 2020; Chen et al., 2023).

The present review aims to provide a comprehensive overview of the current state of adaptively controlled lower limb exoskeleton rehabilitation for stroke treatment. A fundamental prerequisite for realizing an adaptive rehabilitation robot is that the exoskeleton can adaptively perform rehabilitation assessment. Without this premise, the exoskeleton can only be a general rehabilitation device (Tejima, 2001). Therefore, this review systematically surveys the relevant literature on rehabilitation assessment, as well as the human-machine coupling model of exoskeletons in recent decades, and the advances in adaptive control based on motion intention recognition in recent years. Ultimately, the aim of this review is to identify future research directions in this field.

## 2. Rehabilitation assessment

The rehabilitation assessment of stroke survivors is essential throughout treatment (Langhorne et al., 2011). Notably, research conducted by H. J. Appell highlights the need to prevent repetitive mechanical loads on injured skeletal muscles during rehabilitation training, as it can effectively mitigate the risk of secondary damage (Appell, 1990; Appel, 1997). To circumvent adverse effects in the rehabilitation training process, it becomes crucial to modulate the training intensity based on the present condition of the affected muscles. So, The assessment of rehabilitation holds paramount importance in achieving the adaptive control of stroke lower limb exoskeleton rehabilitation robots. Furthermore, the rehabilitation stage following a stroke is intricate, characterized by distinct focal points in different stages (Watson and Quinn, 1998). As a result, the emphasis on rehabilitation training tasks varies in different rehabilitation stages. The reliance on rehabilitation assessment becomes pivotal for adaptive control rehabilitation robots due to the dynamic nature of stroke rehabilitation. Consequently, implementing autonomous rehabilitation assessments within rehabilitation robots becomes imperative to enable real-time adjustments to rehabilitation training tasks (Tejima, 2001).

**Figure 1:**
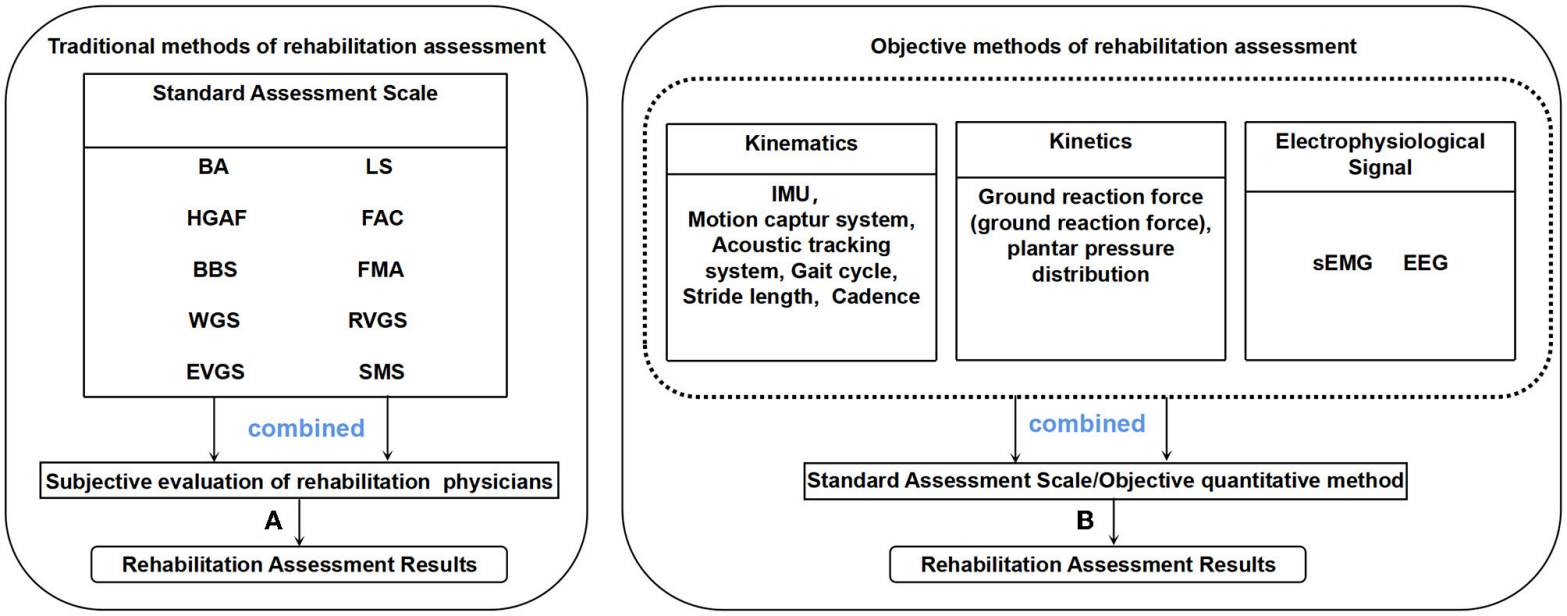
**(A)** Rehabilitation physicians combined with standard evaluation scales to obtain rehabilitation evaluation results, **(B)** Rehabilitation evaluation results are obtained by combining kinematic, kinetic, and electrophysiological signal with standard evaluation scales or other quantitative methods (such as machine learning).

A variety of rehabilitation assessment methods exist for stroke survivors, including functional assessment scales frequently employed in clinical treatment. Additionally, the advancement of technology has led to the emergence of more objective rehabilitation evaluation methods, such as combining kinematic parameters and electrophysiological signals, as depicted in Figure 1.

### 2.1. Traditional methods of rehabilitation assessment

In traditional stroke rehabilitation treatment, rehabilitation physicians need to conduct a rehabilitation assessment on the degree of motor dysfunction of stroke survivors, formulate corresponding rehabilitation treatment plans, or compare recovery conditions later (Colombo et al., 2005). The traditional assessment methods for lower limb rehabilitation in stroke survivors mainly rely on various functional or gait assessment scales (Gonçalves et al., 2022). For example, These standard scales can be scored by judging how well stroke patients complete various tasks so as to achieve rehabilitation assessment. The Wisconsin Gait Scale (WGS) (Rodriquez et al., 1996) uses the Falls Efficacy Scale (FES) (Tinetti et al., 1990) to allow stroke survivors to self-evaluate before and after training and then conduct the Health Status Questionnaire (HSQ) (Radosevich and Pruitt, 1995) test to assess their mental state. This method mainly relies on subjective evaluation. The Brunnstrom Approach (BA) proposed by Brunnstrom (1966), divides the scale into six grades for the limb muscle strength of patients with voluntary movements. Similarly, some rehabilitation physicians also use the LOVETT Scale (LS) to judge muscle strength, which divides stroke survivors into five levels of muscle strength. According to the functional walking ability of stroke survivors, Hughes and Bell (1994) proposed The Hemiplegic Gait Analysis Form (HGAF). And gait parameters divide stroke survivors' standing ability, walking ability, and speed into five levels. There is also a similar scale, Functional Ambulation Classification (FAC), which divides the walking power of stroke survivors and the need for monitoring into 0–6 levels. The Berg balance scale (BBS) evaluates the patient's ability to stand in balance to judge the possibility of falling in stroke survivors (Ganz et al., 2007). The Rivermead Visual Gait Assessment (RVGA) describes 20 kinematic features on a four-point scale and, combined with the subjective evaluation of rehabilitation physicians, is used to evaluate adults with various neurological disorders (Lord et al., 1998). Edinburgh visual gait score (EVGS) uses a 3-point scale to describe 17 kinematic features, combined with the subjective evaluation of rehabilitation physicians to evaluate the recovery of cerebral palsy (Read et al., 2003). The Stroke Mobility Score (SMS) uses a four-point scale to describe six gait characteristics, combined with the subjective evaluation of rehabilitation physicians to explain the recovery of stroke survivors (Raab et al., 2020).

In addition, there is the Fugl-Meyer Assessment (FMA), which is now widely used in the clinical assessment of motor function in stroke survivors, covering five aspects of movement, balance, joint mobility, and pain, and the specific evaluation items reach 113 items. Although many standard scales are used for clinical diagnosis and evaluation, their functions are very similar. The specific selection mainly depends on the subjective wishes of rehabilitation physicians. Moreover, these standard scales are easy for physicians to operate, and the cost of obtaining the rehabilitation status of patients' lower limbs is low. Therefore, it is widely used in clinical practice (Eastlack et al., 1991; Toro et al., 2003). Rehabilitation physicians select the above traditional rehabilitation assessment scales based on comprehensive factors such as the patient's condition, economic conditions, and rehabilitation purposes (for work or life). The schemes adopted can vary from person to person. Often, rehabilitation physicians will choose more than one assessment method for rehabilitation evaluation.

For this reason, more and more kinematic, kinetic and electrophysiological signals, etc. Methods are used to evaluate the rehabilitation of lower limb motor dysfunction. Combined with traditional rehabilitation assessment methods, these methods can realize the adaptive rehabilitation assessment of lower limb exoskeleton robots. Eichler et al. (2018) combined kinematic methods to achieve the same goal by combining a motion capture system with Fugl-Meyer to assess the motor capacity of stroke survivors. Similarly, Wang et al. (2022d) automated assessment of Brunnstrom staging in stroke survivors by surface electromyography. This study demonstrates that subjective assessments by physicians combined with standard scales can be translated into objective rehabilitation assessments of electrophysiological signals. However, in terms of these methods using optical and acoustic sensors, these devices are generally expensive, and the use of space is limited. Such as, when using a visual sensor for rehabilitation assessment, the patient's range of motion cannot exceed the monitoring range of the optical sensor; when using an acoustic tracking system, the patient can only move within the tracking area of the acoustic sensor. Considering the cost of the equipment and the difficulty of the rehabilitation assessment process, in recent years, most scholars have used IMU sensors combined with gait analysis equipment to conduct related research on rehabilitation assessment.

### 2.2. Objective methods of rehabilitation assessment

The main objective rehabilitation assessment methods are based on kinematic, kinetic and electrophysiological signals, etc. (Ferrarello et al., 2013; Mohan et al., 2021; Arheix-Parras et al., 2023). It is necessary to apply accurate rehabilitation assessment methods to lower limb rehabilitation exoskeletons. Lower limb Exoskeletons rehabilitation has excellent application prospects due to the scarcity of professional rehabilitation physician resources (Gupta et al., 2011). Taking China as an example, a rehabilitation physician often needs to take care of several patients, and the lower limb rehabilitation exoskeleton can significantly reduce the unnecessary repetitive work of rehabilitation physicians. The lower limb rehabilitation exoskeleton can perform various rehabilitation training tasks and use multiple sensors on the lower limb rehabilitation exoskeleton to assess the rehabilitation of stroke survivors at a low cost. This not only reduces the error caused by the combination of doctors' artificial judgment and various evaluation scales but also avoids the duplication of labor of medical staff. This helps rehabilitation physicians to focus more on the formulation of rehabilitation programs. In addition, these relevant parameters used for rehabilitation assessment can also be used for motion intention recognition of exoskeleton rehabilitation robots. The obtained evaluation results are conducive to constructing a better adaptive control model for the lower limb rehabilitation exoskeleton combined with the patient's current recovery and exercise ability. The objective rehabilitation assessment methods are overview in Table 1.

**Table 1:** Overview of objective rehabilitation assessment.

**Paper**	**Tools**	**Limb**	**Method**	**Assessment objectives**
Eichler et al. (2018)	3D cameras, FMA	Whole body	SVM^1^, SDT^2^ and RF^3^	Distinguish between the three known stroke severity.
Wang et al. (2022d)	sEMG, Brunnstrom	Forearms	Ensemble Learning	Brunnstrom stage automatic evaluation for stroke survivor.
Postolache et al. (2015)	Doppler radars, Gait sensors	Legs, Feet	Wavelet multiresolution analysis	Assess the instantaneous velocity of the leg swing.
Park et al. (2020)	IMU, NIHSS and MRC	Wrists, Ankles and Feet	Ensemble algorithm, SVM	Automatically assess stroke survivor' NIHSS grades and MRC scores.
Zhao et al. (2017)	IMU, Pressure sensors	Knees, Ankles and Feet	Inequality-constrained Zero Velocity Updates-aided Inertial Navigation System algorithm	Foot Angle for Assessing Extension-Flexion Movement in Stroke survivors.
Guo et al. (2019)	sEMG, A State-Space EMG Model (Han et al., 2015)	Upper limbs	Bayesian classifier	Distinguishing stages of stroke rehabilitation.
Zeng et al. (2017)	EEG	Corresponding brain area of the lesion	Mean-NLSD^4^, PSD^5^	Assess stroke recovery.
Wang et al. (2022b)	sEMG, EEG	Corresponding brain area of the lesion, Dorsal interosseous	PTE^6^	Using brain network features and cortical muscle coupling values to distinguish rehabilitation stages.
Bervet et al. (2013)	EMG, Cameras and Pressure sensors	Feet, Legs and Heels	KeR-EGI^7^	Gait recognition on healthy subjects.
Meng et al. (2021)	sEMG, IMU	Weist, Forearms, Legs and Ankles	LOSOCV^8^	Mobility capacity in a stroke survivor.
Spanos et al. (2023)	Cameras, G.A.I.T	Hip, Legs and Ankles	KNN^9^	Gait recognition on healthy subjects.
Wang et al. (2020)	Cameras, IMU	Legs and Ankles, Feet	INI^10^	judge the recovery of each leg.

1 SVM, Support Vector Machines;

2 SDT, Single Decision Tree;

3 RF, Random Forest;

4 Mean-NLSD, Mean-Nonlinearly Separable Degree;

5 PSD, power Spectral Density;

6 PTE, Phase Transfer Entropy;

7 KeR-EGI, Kerpape-Rennes EMG-based Gait Index;

8 LOSOCV, Leave-One-Subject-Out Cross-Validation;

9 KNN, K Nearest Neighbor;

10 INI, IMU-based gait normalcy index.

#### 2.2.1. Kinematic, kinetic-based objective rehabilitation assessment method

In recent decades, with the development of technology, more and more studies have evaluated lower limb functional rehabilitation through kinematic methods. For example, Postolache et al. (2015) assessed the gait ability of patients using a wheeled walker by microwave Doppler radar. The Inertial Measurement Unit (IMU), which can detect the patient's speed, acceleration, joint angle, and other motion parameters, can be used for rehabilitation evaluation (Ahmad et al., 2013). The IMU sensor has two significant advantages: First, the sensor is cheap and can be widely used in hospitals (Maetzler et al., 2013). Second, it is easy to wear, can be used in various scenarios by patients, and the detection process is simple (Del Din et al., 2016). Park et al. (2020) collected the kinematic parameters of the subject's limbs through the IMU. They automatically evaluated the NIH Stroke Scale/Score (NIHSS) (Williams et al., 2000) grade and Medical Research Council (MRC) (Paternostro-Sluga et al., 2008) score of stroke survivors through machine learning. However, IMU also has limitations. The IMU can only detect the patient's motion state, not the patient's posture. When the human body joints move, the joint vibration angiography is relatively large, interfering with the results (Wang et al., 2022a). Therefore, in recent years, some scholars have combined IMU sensors with gait recognition equipment. Zhao et al. (2017) used the IMU-based gait analysis method to evaluate the rehabilitation of lower limb dysfunction. The Gait Assessment and Intervention Tool (G.A.I.T) combines motion capture equipment to conduct rehabilitation assessments of stroke survivors from 45 independent gait poses combined with rehabilitation physicians' experience (Daly et al., 2009; Ferrarello et al., 2013) Some scholars also use optical sensors to conduct rehabilitation assessments through motion capture methods (Eichler et al., 2018). Wang et al. (2020) improved the above method, using IMU, gait recognition, and motion capture system, and other equipment to judge the recovery of each leg from three spatiotemporal parameters and six kinematic features, and proposed an IMU-based gait normalcy index (INI) rehabilitation assessment method. Or use an acoustic tracking system, such as Maki et al. (2012) developed an ultrasonic stride measurement system, which uses ultrasound to locate the position of the sensors of the left and right feet to achieve step distance measurement. The evaluation process of these kinematic methods is simple, and the rehabilitation evaluation can be completed only by the patient walking on the experimental site relying on the corresponding sensors. Based on objective rehabilitation assessment methods, this can effectively reduce the burden of repetitive motion in stroke survivors.

#### 2.2.2. Electrophysiological signals-based objective rehabilitation assessment method

Standard electrophysiological signals used in evaluating movement disorder rehabilitation include surface electromyographic (sEMG) signals and electroencephalogram (EEG) signals (Frigo and Crenna, 2009; Cohen, 2017). For example, Guo et al. (2019) collected sEMG of patients in different rehabilitation stages, classified and identified sEMG through machine learning methods, and verified that sEMG could identify different rehabilitation stages. However, there are still many problems with this method. For ordinary people, the period of coordinated muscle movement in normal gait is fixed (Huang et al., 2012). In contrast, the regularity of muscle activation in stroke survivors is poor (Dai et al., 2004), and even specific muscles cannot be activated so the evaluation results will differ. In addition, with the increase in exercise in lower limb rehabilitation training, muscle fatigue will occur, resulting in decreased muscle strength (Riley and Bilodeau, 2002). All of the above reasons make rehabilitation assessment using sEMG difficult.

Therefore, in recent years, people have increased research on EEG. EEG records the activity of neurons in the cerebral cortex. Stroke causes brain lesions in patients, which directly drives the EEG of stroke survivors to be different from ordinary people (Finnigan and van Putten, 2013). This difference is used for a rehabilitation assessment. Zeng et al. (2017) evaluation of stroke rehabilitation by detecting non-linear EEG of patients. Due to the complexity of the recording process of EEG signals, the acquisition requires a high degree of concentration. It is susceptible to interference, which restricts the wide application of EEG signal rehabilitation assessment. And Wang et al. (2022b) collected EEG and sEMG to construct a brain function network and corticomuscular PTE, using brain network features and cortical muscle coupling values to distinguish rehabilitation stages. This study provides a basis for the internal mechanism of stroke hemiplegic patients' rehabilitation from the perspective of physiological signals. Still, the recovery period of selected stroke survivors is only four weeks, which is not comprehensive for the entire rehabilitation period. Physiological electrical signals are collected directly from the human body and are not affected by subjective factors. Rehabilitation assessments can be performed by combining signal processing and related algorithms. There are also deficiencies in electrophysiological signals. Due to the characteristics of electrophysiological signals and the relatively complicated acquisition process, they impact rehabilitation assessment.

#### 2.2.3. Multimodality-based objective rehabilitation assessment method

According to the advantages and disadvantages of kinematic methods and electrophysiological signals, more and more scholars combine them for stroke rehabilitation assessment. For example, Bervet et al. (2013) uses the fusion of EMG and gait detection to judge the overall gait performance of stroke survivors through 7 kinds of EMG gait patterns. Meng et al. (2021) used sEMG and IMU to assess stroke survivors' mobility by adopting the Leave-One-Subject-Out Cross-Validation (LOSOCV) technique, but with a small sample size for stroke survivors. These related studies can be applied to the adaptive lower limb rehabilitation exoskeleton robot to better build an adaptive control model according to the patient's current recovery situation and exercise ability.

### 2.3. Development of rehabilitation assessment methods

As indicated above, the rehabilitation assessment of stroke survivors has evolved from the previous traditional approach, which solely relied on rehabilitation assessment scales combined with subjective evaluations by rehabilitation physicians, to the current integration of various kinematic parameters, kinetic parameters, electrophysiological parameters, or novel rehabilitation assessment scales. It is the transition from complete observation to instrumental measurement. We have gathered and summarized the literature about rehabilitation assessments conducted over the past three decades. The methods employed in these assessments encompass observational, instrumental, kinematic, kinetic, electrophysiological parameters, and integrative approaches.

Between 1993 and 2009, rehabilitation assessments were mainly implemented with standard scales and observational methods. For instance, the Physician's Rating Scale (PRS) (Koman et al., 1993) assesses the severity of cerebral palsy by recording six gait characteristics using a multi-point scale by rehabilitation physicians. The PRS-Based Observational Gait Scale (RPS-OGS) (Boyd and Graham, 1999; Boyd et al., 1999) builds upon this method by expanding the assessment to eight gait characteristics for each leg. Another scale, The Wisconsin Gait Scale (WGS) (Rodriquez et al., 1996), employs a weighted multi-point scale to record 14 gait characteristics for each leg, reflecting the recovery progress of hemiparesis. Additionally, previously mentioned scales like RVAG and EVGS are utilized. Observational Gait Analysis (OGA) (Kawamura et al., 2007) employs a three-point scale to score and describe ten kinematic features, assessing the degree of cerebral palsy. The Salford Gait Tool (SF-GT) (Toro et al., 2007) utilizes a five-point scale to achieve and describe 18 kinematic features, evaluating the degree of cerebral palsy. The Observational Gait Scale (OGS) (Araújo et al., 2009) further expands the evaluation content by incorporating 24 kinematic features and the observational assessment by rehabilitation physicians to assess the severity of cerebral palsy. These methods are mainly based on observational methods or weak alternatives to instruments with a certain degree of subjectivity. In the following research, the rehabilitation assessment method gradually became instrumentalized, making the rehabilitation assessment results more objective and accurate.

More and more researchers use instruments to collect the patient's kinematics, kinematic and electrophysiological features and combine computer and statistical methods to evaluate the patient's rehabilitation rather than pure observation. As introduced in Section 2.2. In recent years, multimodality rehabilitation assessment methods have become a hot topic. The development trend of rehabilitation assessment instrumentation also makes the adaptation assessment of exoskeleton robots possible (Shi B. et al., 2019).

Despite the inherent subjectivity of observational methods based on standardized assessment scales, they are still widely used in clinical practice due to their simplicity, cost-effectiveness, and other advantages (Toro et al., 2003; Rathinam et al., 2014). Simultaneously, with the continuous advancement of technology, there is an increasing demand for the application of exoskeleton robots, which has led to rapid development in instrument-based gait analysis methods. Due to their accuracy and reliability, these methods have now become the preferred gold standard for clinical decision-making and rehabilitation instrument researchers (Wren et al., 2020).

## 3. Human-machine coupling model of lower limb exoskeleton

The lower limb rehabilitation exoskeleton robot is a wearable bionic robot that can be coupled with man-machine (Zhou J. et al., 2021). Combined with various sensors, a kinematic model is established through kinematic analysis to realize impedance/admittance/compliance control (Scherzinger et al., 2017; da Silva et al., 2020). Finally, the high-efficiency actuators apply external force to the patient's lower limbs to assist the patient's movement so that the patient can move independently (Windrich et al., 2016). Such as, assist stroke survivors in going up and down the stairs independently or walking on flat ground, etc, to achieve the purpose of rehabilitation training (Alcobendas-Maestro et al., 2012; Guizzo and Deyle, 2012; Chen et al., 2016; Wang et al., 2022c).

In this section, the human-machine coupling mode of the lower limb exoskeleton robot that has been put into production and the control model of the lower limb exoskeleton based on efficient actuators will be introduced.

### 3.1. Existing human-machine coupling models of lower limb exoskeletons

Currently, more and more studies use lower limb exoskeleton rehabilitation robots to assist patients in rehabilitation training and reconstruct the patient's motor function (Zhou X. et al., 2021). Due to the limited mobility and balance ability of stroke survivors, it is necessary to pay special attention to the safety of stroke survivors during rehabilitation training to prevent them from falling during exercise and causing secondary trauma (Zhou J. et al., 2021). Therefore, different lower limb rehabilitation exoskeleton robots can be selected according to the patient's exercise balance ability (Wang et al., 2022c).

At present, the standard lower limb rehabilitation exoskeleton robots on the market are:

1. LOKOMAT, Stroke survivors wear lower limb exoskeleton robots and protective straps and perform autonomous movements on the platform treadmill. LOKOMAT can also provide adaptive support for stroke survivors according to their leg strength (Duschau-Wicke et al., 2008).
2. REX, It is a lower limb exoskeleton rehabilitation robot with self-balancing ability, which provides external support for stroke survivors and assists them in standing. The stroke survivor joystick controls REX movement. Nathan et al. (2021) combine brain-computer interface technology with REX and use EEG to control REX movement.
3. ReWalk, The wearable lower limb exoskeleton device incorporates crutches and therefore requires the patient to have some mobility and balance (Zeilig et al., 2012). When in use, the patient's hands are supported by crutches, and then they try to take a step, and the sensor detection data at ReWalk's hip joints and joints drive the exoskeleton's movement through actuators, simulating the natural movement of human legs, thereby driving stroke survivors to move (Nathan et al., 2021).

**Figure 2:**
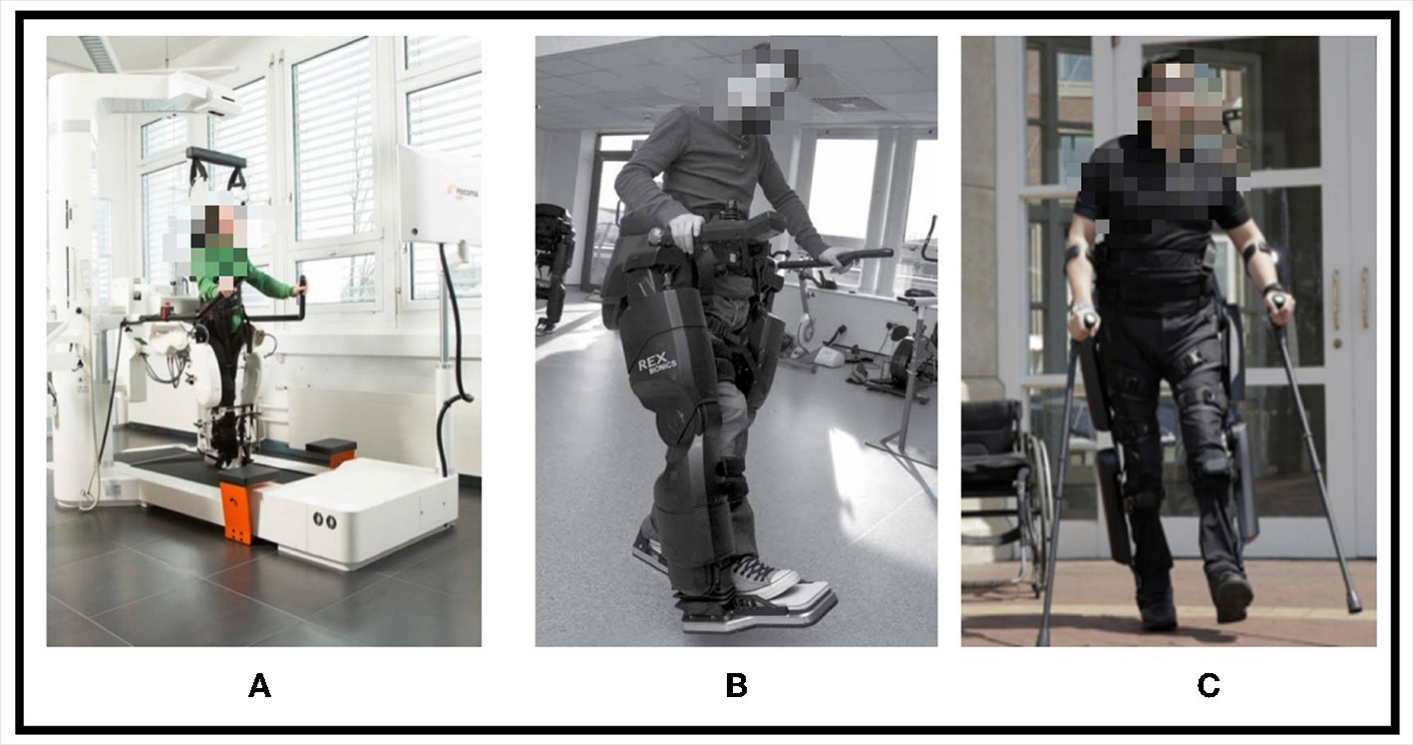
Three types of lower limb exoskeletons, **(A)** LOKOMAT, **(B)** REX, **(C)** ReWalk.

These three types of lower limb exoskeletons are shown in Figure 2. The LOKOMAT provides enhanced safety for stroke survivors through protective straps. It incorporates multiple IMU sensor units to recognize the movement intentions of stroke survivors, enabling them to participate actively in training rather than being passive recipients, which is beneficial in motivating stroke survivors to engage actively in rehabilitation exercises (Marchal-Crespo and Riener, 2022). The LOKOMAT therapy is currently regarded as a promising method for restoring functional walking and improving motor abilities in stroke survivors (Alashram et al., 2021). In addition, lower limb exoskeletons that can be used for rehabilitation also rely on efficient actuators and control algorithms.

### 3.2. The lower limb exoskeleton model is based on actuators

The actuator is an essential part of the automatic control system (Hussain et al., 2021). It receives the control signal of the controller and completes the corresponding functional output. Actuators are divided into active and passive actuators according to whether they consume energy. Active actuators consume energy, such as hydraulic, electric, and pneumatic, and passive actuators, such as springs and dampers, do not consume power (Ham et al., 2009). When performing lower limb movements, more torque is generated than other body parts. Each joint has multiple Degrees of Freedom (DoF), and the joints of the lower limbs of the human body mainly include hip joints, knees, and ankles (Gholap et al., 2022). The specific movement modes and DoF of the lower limb joints are shown in Table 2. Therefore, the combination of lower limb exoskeleton actuators that rely on joint motion has hip-knee, knee-ankle, and hip-knee-ankle (Sun et al., 2020, 2021).

**Table 2:** The specific movement modes and DoF of the lower limb joints.

**Joints**	**DoF**	**Movement**
Hips	3	Internal-external rotaion; Abduction-adduction; Flexion-extension.
Knees	2	Flexion-extension; Rotaion.
Ankles	3	Plantar flexion-dorsiflexion; Abduction-adduction; Eversion-inversion.

Generally, a lower limb rehabilitation exoskeleton robot requires multiple DoF actuators to cooperate, and actuator combinations will be used according to lower limb exoskeleton kinematic models (Pamungkas et al., 2019). This section is illustrated in terms of several actuator-specific models.

#### 3.2.1. Exoskeleton model based on pneumatic actuators

Głowiński and Ptak (2022), based on the relevant measurement parameters of the human body, the geometric model of the pneumatic exoskeleton was established, and the dynamic gait parameters were analyzed. The designed pneumatic exoskeleton actuator combination includes hip joints, knees, and ankles, which can generate the physiological torque required for human joint movement. The lower limb rehabilitation exoskeleton is a human-machine coupling device, so the device's safety is paramount. For pneumatic actuators, the pressure limit of each actuator needs to be strictly determined before use, which can effectively ensure the safety and comfort of humans and exoskeletons (Moreno et al., 2018). Therefore, the lower limb exoskeleton rehabilitation robot based on pneumatic actuators has been one of the popular choices for research in recent years. The pneumatic exoskeleton model also has limitations. First, it is not absolutely safe. The elastic potential energy stored in the pneumatic actuator may be released suddenly, and the resulting reaction force may hurt the human body (Grosu et al., 2017). Moreover, this model did not further study the compensation mechanism of the axis misalignment of the exoskeleton and the patient's knee joint (Bessler-Etten et al., 2022).

#### 3.2.2. Exoskeleton model based on hydraulic actuators

Chen et al. (2021) proposed a 3-DOF precise interactive controller model for an underactuated hydraulic exoskeleton driven by hydraulic actuators. This model transforms the 3-DOF underactuated system in the joint space into a 2-DOF full-actuated system in the Cartesian coordinate system and solves the related problems of high-order nonlinearity, parameter uncertainty, and modeling errors in the hydraulic actuator system. The three-level interactive force controller can realize motion intention inference, human body motion tracking, and hydraulic output capability tracking. Hydraulic-type actuators can provide mighty power and adequate human body support (Zhu et al., 2017). However, hydraulic actuator models require various pumps and valves, which increase the volume and weight of the exoskeleton, which is inconvenient for stroke survivors to use in their daily lives (Scheidl, 2017).

#### 3.2.3. Exoskeleton model based on motor actuators

Compared with the models built with the above two actuators, the exoskeleton robot based on the motor drive is more popular because of its convenient control process (Gholap et al., 2022). Feigin et al. (2022) designed the exoskeleton motion model based on the position control of direct current (DC) servo motors. The exoskeleton actuator combination includes a hip-knee-ankle, and 6 DC motors are used. The model adjusts and guarantees the angular position of the motor shaft through a closed-loop feedback control system, obtains the joint dynamics parameters of human cyclic gait through the IMU sensor, and uses PID (Process Identification) control (Vanchinathan and Selvaganesan, 2021) to realize the stable control of the exoskeleton.

#### 3.2.4. Exoskeleton model based on other actuators

In addition to the basic actuator exoskeleton models mentioned above, there are linear elastic actuator exoskeleton models, parallel elastic actuator exoskeleton models, etc (Zhu et al., 2014; Beil et al., 2015). Models based on different actuator structures are the most basic exoskeleton models, which can complete specific mechanical actions. Lower limb rehabilitation exoskeletons need to adapt to multiple complex situations to meet the requirements of stroke survivors for various rehabilitation tasks and assist them in daily life (Mohammed et al., 2012).

Aljarah et al. (2023) have introduced a Variable Stiffness Actuator (VSA) for Knee Exoskeletons. It comprises a torsion spring, a motor, a linear actuator, and a force contact roller (FCR). The stiffness of the brake is determined by the spring's stiffness, the diameter of the spring base, and the diameter of the FCR. The researchers employed the actuator using the Pareto grid-searching method. This method optimizes the weighted objective function and avoids restrictive standardization techniques. Finally, the operating parameters of the knee exoskeleton were optimized and verified.

Nunes et al. (2020) designed a bio-inspired controller via elastic actuators based on the concept of motor primitives. Momentum-based disturbance observer with extended Kalman filter to extract motor primitives. A control algorithm then analyzes the gait cycle to test the actuator's performance in motion. The results show that the exoskeleton's auxiliary compensation force to the knee joint is insufficient when the motion torque is too small.

In the early days of related research on exoskeleton adaptive control, Jatsun et al. (2015) used the current information of the motor iron core to evaluate the load of the exoskeleton and then adaptively change the action of the exoskeleton, which realized the adaptive control of exoskeleton. However, Jatsun et al. (2015) follow-up research found that for the application of the rehabilitation exoskeleton, this exoskeleton's movement is blunt, which is not conducive to the rehabilitation training of stroke survivors. Therefore, the adaptive lower limb rehabilitation exoskeleton robot based on motion intention recognition has become the research focus (Long et al., 2023). To cooperate with the related algorithms of motion intention recognition, multi-source information are also needed for motion intention detection (Zhang et al., 2022).

## 4. Motion intention recognition and modeling of adaptive lower Limb exoskeleton

With the development of science and technology, Artificial Intelligence (AI)-related technologies have become more research hotspots today. AI-based lower limb exoskeleton control models are no exception (Vélez-Guerrero et al., 2021; Halder and Kumar, 2023), such as artificial neural networks, adaptive algorithms, and other mixed AI techniques.

Among them, The adaptive lower limb rehabilitation exoskeleton robot based on motion intention recognition uses different method to obtain relevant signals of the current motion and through the identification and extraction of the present motion state (Long et al., 2023). In this way, the next movement to be completed can be predicted, and the motion intention of stroke survivors can be decoded (Zhang et al., 2022). Finally, an adaptive control model is constructed to control the exoskeleton actuator.

The following introduces the methods based on motion intention recognition combined with artificial neural networks, adaptive control algorithms and other fusion AI methods in recent years. Currently, the lower limb exoskeleton rehabilitation robot mainly builds an adaptive control model through kinematics, kinetics, electrophysiological signal, and multimodal fusion (Sun et al., 2022; Masengo et al., 2023).

**Figure 3:**
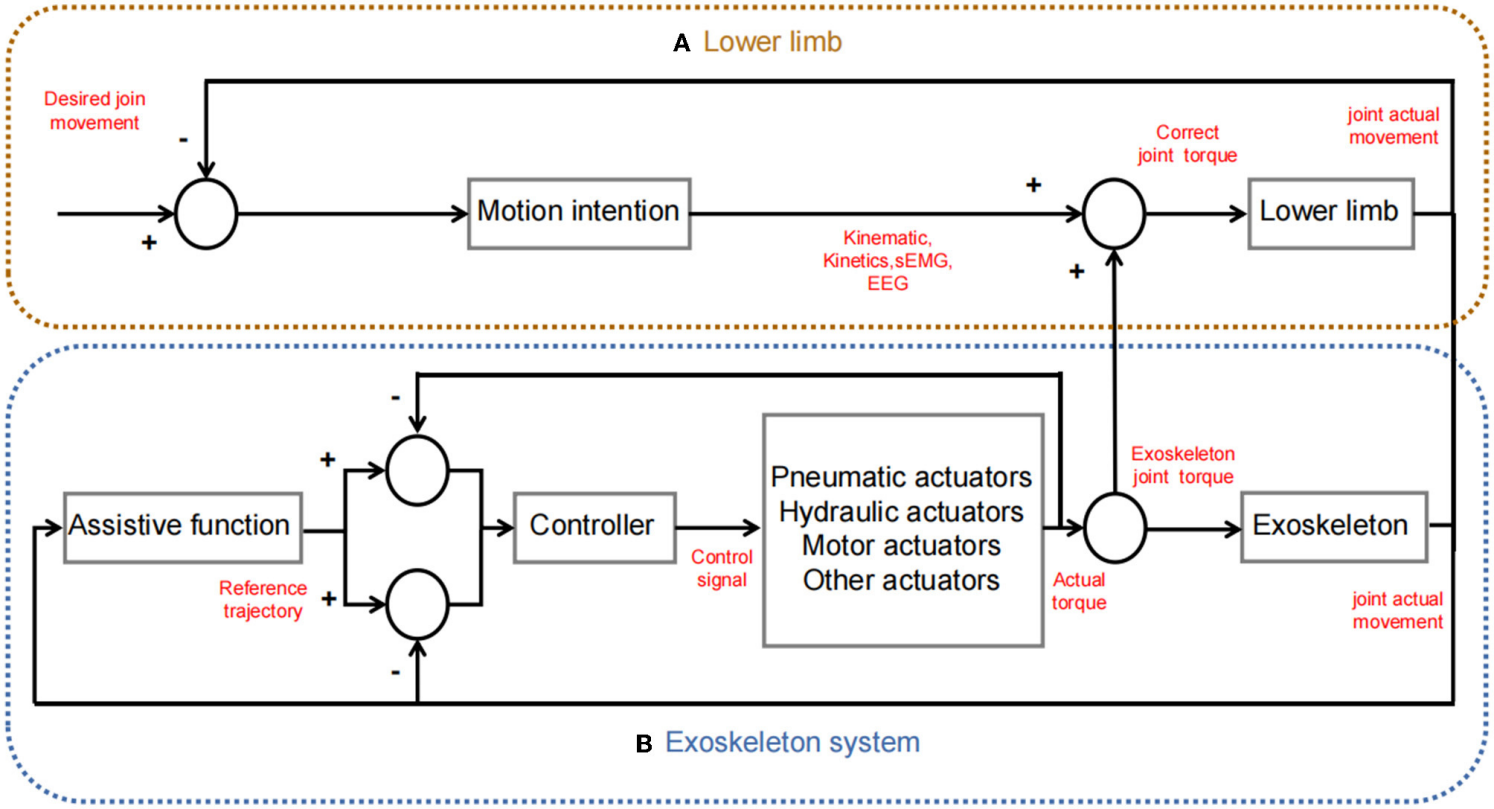
Scheme of common lower limb exoskeleton human-machine cooperative control. **(A)** Lower limb, provides kinematics, kinetics, and electrophysiology information. **(B)** Exoskeleton system, the movement trajectory is planned through functions; the controller drives the actuator to generate corresponding torque; and human-machine cooperation (the power generated by the actuator assists the movement of the lower limbs).

The overview of the adaptive exoskeleton control model based on motion intention recognition introduced in this paper is shown in the Table 3. Scheme of common lower limb exoskeleton human-machine cooperative control is shown in Figure 3. The Human-exoskeleton coordinated control strategy mentioned in this chapter are shown in Table 4.

**Table 3:** Overview of the adaptive exoskeleton control model based on motion intention recognition.

**Paper**	**Tools**	**Main research**	**Method**	**Pourpose**
Zhu et al. (2020)	IMU	Perception	Deep CNN	Accurately identify distinct strategies, including level walking, stair ascent/descent, and uphill/downhill walking, and accuracy achieves 97.64%.
Ding et al. (2018)	iIMU	Perception	Local search windows, Fixed thresholds	Achieved a minimal time delay and small computational burden.
Chen et al. (2022)	Kinematic	Perception, Control	NFO	Improves the precision and speed of intent recognition, enhancing adaptive control performance for the exoskeleton.
Zhang et al. (2019)	Kinematic	Control	Hierarchical Lyapunov	The model is satisfactory in tracking performance and interaction torque reduction.
Sánchez Manchola et al. (2019)	IMU, Plantar pressure	Perception	TB, HMM	HMM has better performance in terms of time error and goodness index.
Ma et al. (2020)	VICON	Perception, Control	DMP	Constructed a gait parameter model.
Hua et al. (2019)	Kinematic, Plantar pressure	Perception, Control	DNNs, GA_PSO and ANFIS	Achieved intent recognition accuracy of 99.7% and achieved adaptive control.
Wang et al. (2022d)	Tension sensors, Photoelectric sensors and IMU	Perception	PNN^11^, SVM-RBF^12^	Classify and identify the behavioral state of the user during rehabilitation training.
Liu et al. (2022)	sEMG	Perception	ML-TCN, ML-LSTM	Gait cycle phase recognition; Compared the effect of the TCN model and the LSTM model.
Guo et al. (2020)	sEMG	Perception, Control	Lw-CNN, SVM	Recognize upper-limb motion intents; Comparing the recognition effect of the Lw-CNN model and SVM.
Choi and Kim (2019)	EEG	Perception	CSP^13^; LDA^14^; MTM-TSC	Gait detection.
Lou et al. (2019)	FAC, IMU	Perception	Sliding Window Algorithm^15^; QDA^16^	Recognizing the gait phase.
Liang et al. (2018)	Motion capture devices	Perception, Control	LSTM	The motion intention of the healthy side of the stroke survivors is used to plan the trajectory of the exoskeleton on the hemiplegic side.
Novak et al. (2013)	IMU, Plantar pressurer	Perception	supervised machine learning	Have high accuracy in detecting patterns of gait initiation and gait termination.
Song et al. (2020)	IMU, Plantar pressurer; VICON	Perception	Multi-layer BP neural network	15 common lower limb exoskeleton gait patterns and postures identified.
Du et al. (2018)	sEMG, Plantar pressurer	Perception, Control	SMILC	The model can effectively reduce chattering in sliding mode control and excellently achieve the tracking of the rehabilitation robot's reference trajectory.
Gui et al. (2019)	sEMG, Joint torque	Perception	RBFNNs	Joint motion prediction.
Zheng et al. (2020)	EEG, Kinematic and Plantar pressure	Perception	CCA	Motion intention recognition through multimodal models.
Al-Quraishi et al. (2021)	EEG, sEMG	Perception	DCA	A multi-modal control model is constructed to recognize lower limb movement intention.
Amiri et al. (2019)	Kinematic	Control	IMRAC	This modle can be used in lower limb exoskeleton as a gait training robot for rehabilitation purpose.

1 PNN, Probabilistic Neural Network;

2 RBF, Radial Basis Kernel Function;

3 CSP, Common Spatial Pattern;

4 LDA, Linear Discriminant Analysis;

5 SWA, Sliding Window Algorithm;

6 QDA, Quadratic Discriminant Analysis.

**Table 4:** Comparison of Human-exoskeleton coordinated control strategy.

**Paper**	**Control approach**	**Advantages**	**Challenges**
Amiri et al. (2019)	IMRAC	The comparison between the results of the IMRAC and conventional MRAC, shows the IMRAC converged faster and consumed less computational time than MRAC.	Lack of testing under disturbing conditions or in different environments.
Liang et al. (2018)	Based on LSTM.	Comparison with statistical regression methods based on LSTM, is stable, disturbed only by missing data and outliers, and has better inter-individual adaption.	Adaptability to a large number of subjects was not tested.
Zhang et al. (2019)	Based on hierarchical Lyapunov.	Compared with PD control, the moment of human-computer interaction can be minimized by setting appropriate controls.	There will be a delay in lower layer mode switching.
Chen et al. (2022)	Based on NFO.	It has faster calculation speed and recognition accuracy than GA and PSO algorithms.	The stability is easily affected by the parameters of the PD controller.
Ma et al. (2020)	Based on DMPs.	Trajectory features are extracted from sample trajectories, and new trajectories are constructed.	It is impossible to construct a general model, and information such as the subject's height and weight needs to be considered.
Guo et al. (2020)	Based on Lw-CNN.	Compared with SVM, the intent recognition speed is faster, and the real-time control is better.	The recognition accuracy is affected by the window size, and the accuracy decreases significantly when the sliding rate decreases.
Hua et al. (2019)	GA_PSO algorithm.	Compared with the sensitivity amplification control (SAC), the human-robot interaction is reduced by 0.6%, which reduces the burden of human-robot collaborative motion.	Gait conversion and label categories are not subdivided, and the recognition conversion efficiency is not high.
Du et al. (2018)	Variable impedance controller and SMILC.	Compared with PD iterative learning control algorithm (PDILC), the tracking error of SMILC is more minor, and the control system is more stable.	SMILC is prone to jitter when the state trajectory reaches the sliding mode surface.

### 4.1. Adaptive model of lower limb exoskeleton for motion intention recognition based on kinematic and kinetics

**Figure 4:**
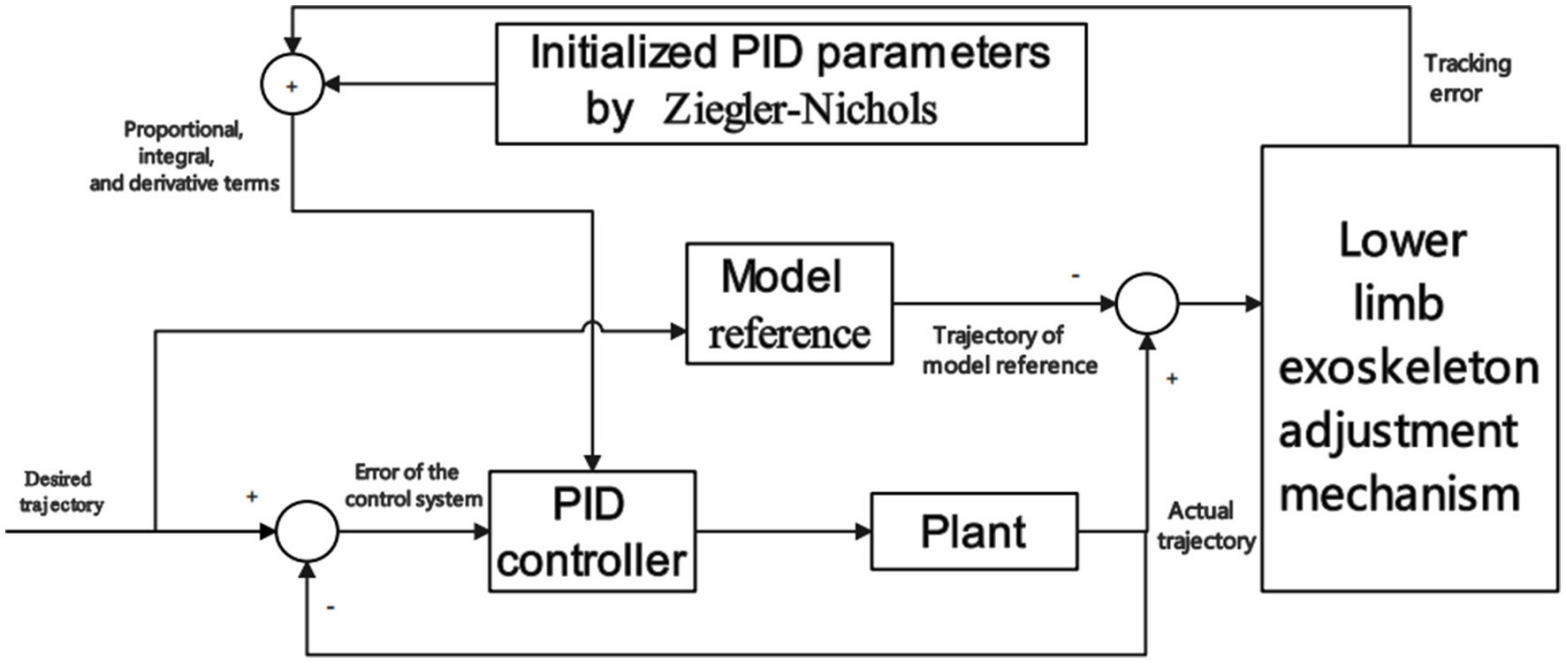
Scheme of IMRAC. IMRAC includes a plant, a lower limb exoskeleton adjustment mechanism controller parameters, and a model reference.

The kinematic signals for motion intention recognition include the acceleration of the current motion of the human body, motion speed, limb motion trajectory, joint angle, joint torque, and plantar pressure. The IMU can collect human kinematics parameters such as acceleration, velocity, and angular velocity signals for lower limb motion state recognition (Hamdi et al., 2015). Amiri et al. (2019), Using a traditional cybernetics approach, by kinematic, initialized Model Reference Adaptive Control (IMRAC) is used to tune a Proportional-Integral-Derivative (PID) controller of a lower limb exoskeleton. The model performs well in real-time but was not tested in a test environment with disturbances. Scheme of IMRAC is shown in Figure 4.

Lou et al. (2019) relied on the FAC to classify the patient's exercise capacity into 0-6 grades, and patients with grades 3–4 were selected for the trial. That study mainly identified stroke survivors' standing and gait phase state in the gait cycle through the relevant data collected by IMU. They also concluded that the more severely disabled, the lower extremities of the stroke survivors, the more significant the proportion of the total gait cycle that the stance phase occupies. Although this study did not further construct the adaptive control model of the lower limb exoskeleton, it still has a specific role in promoting the research on the exoskeleton robot for stroke lower limb rehabilitation.

Zhu et al. (2020) have introduced a motion intent recognition method for soft exoskeletons based on inertial sensors. This method exhibits exceptional performance in diverse and intricate scenarios. By leveraging an Inertial Measurement Unit (IMU) to gather kinematics, they utilize a deep Convolutional Neural Network (CNN) to accurately identify distinct strategies, including level walking, stair ascent/descent, and uphill/downhill walking. The recognition accuracy achieves 97.64%. Moreover, the latency associated with switching between different recognition modes accounts for a mere 23.97% of the gait cycle duration.

**Figure 5:**
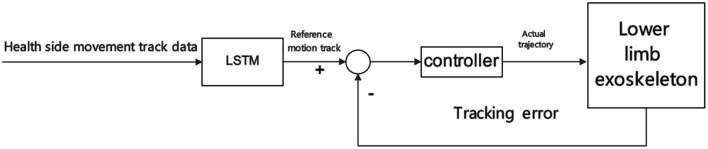
LSMT-based control scheme.

Liang et al. (2018) proposed a novel control method. They explained the synergy between human limbs through LSTM, expecting to collect a large amount of gait data of ordinary people through wearable motion capture devices. Then use LSTM to analyze the motion data of one side of the healthy person's limb, and then compare the motion data of the other side of the stem, extract synergy from the data, build an adaptive control model of the exoskeleton, and finally adapt online to stroke survivors at different rehabilitation stages, and achieve through The motion intention of the healthy side of the stroke survivor is used to plan the trajectory of the exoskeleton on the hemiplegic side. This study has successfully achieved cooperative control on a small amount healthy subjects. Still, there is a lack of relevant experimental data for stroke survivors, which is one of the relevant research issues of the exoskeleton adaptive control model based on motion intention recognition. When building a control model, healthy people are often used as subjects. While the model is verified, it is also tested by healthy people wearing exoskeletons. LSTM-based control scheme is shown in Figure 5. Therefore, whether the adaptive control model established in the study applies to stroke survivors has not been verified, nor can the problems of stroke survivors using exoskeletons for rehabilitation training be found.

Novak et al. (2013) obtained kinematic parameters through IMU and pressure-sensitive sensors, constructed an adaptive control model based on kinematic parameter identification, and verified the exoskeleton model through healthy subjects, which high accuracy in detecting patterns of gait initiation and gait termination, but also lack specific clinical trials.

**Figure 6:**
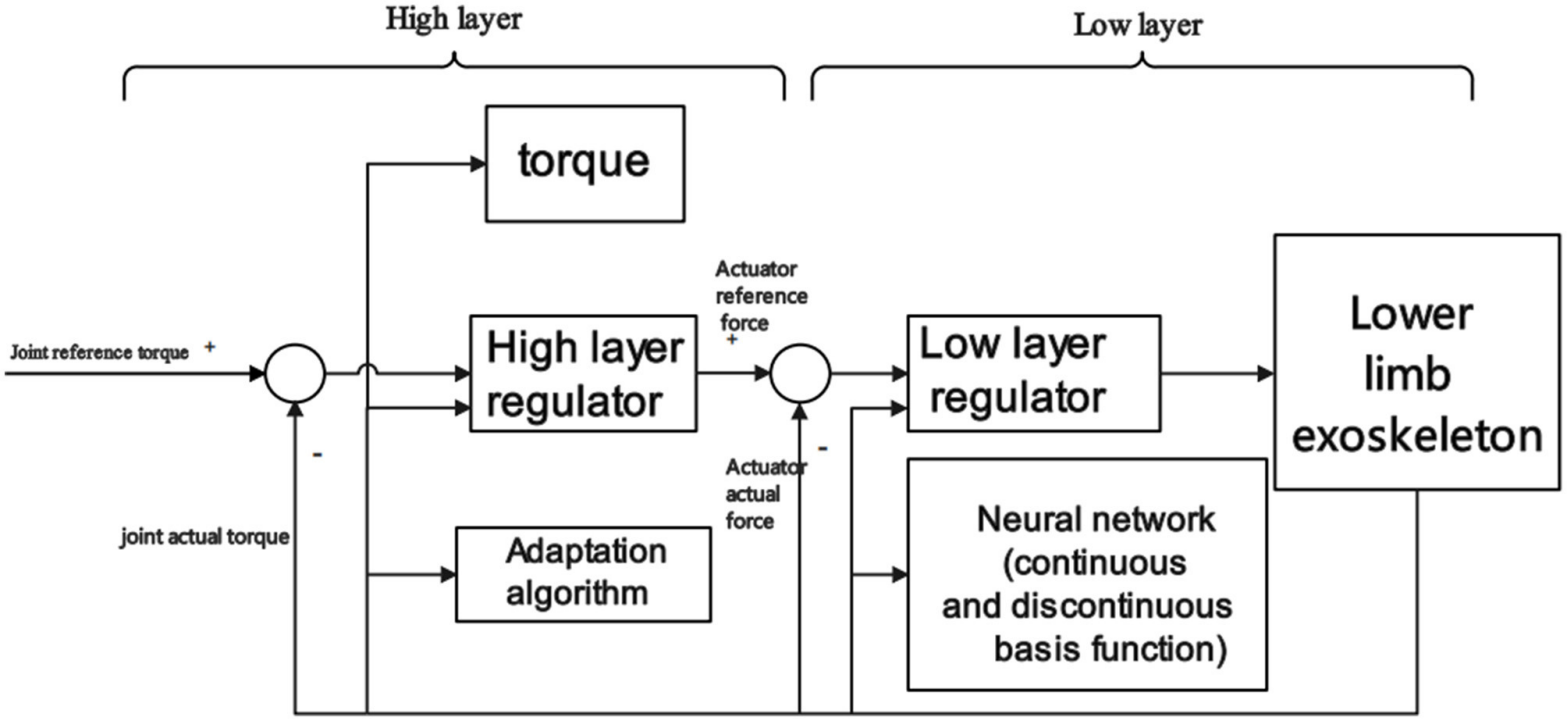
Scheme of adaptive control model based on hierarchical Lyapunov.

Zhang et al. (2019) constructed a cascaded adaptive control model based on hierarchical Lyapunov for joint angle position tracking objective. First, a Lyapunov-based backstepping regulator was designed to adaptively estimate the parameters and friction in the higher-level structure. Then in the lower layer, The hydraulic actuator is controlled by a Lyapunov-based neural network to realize lower limb exoskeleton adaptive control. These control models have not been tested for different rehabilitation tasks, and clinical trials for specific conditions are lacking. Scheme of adaptive control model based on hierarchical Lyapunov is shown in Figure 6.

**Figure 7:**
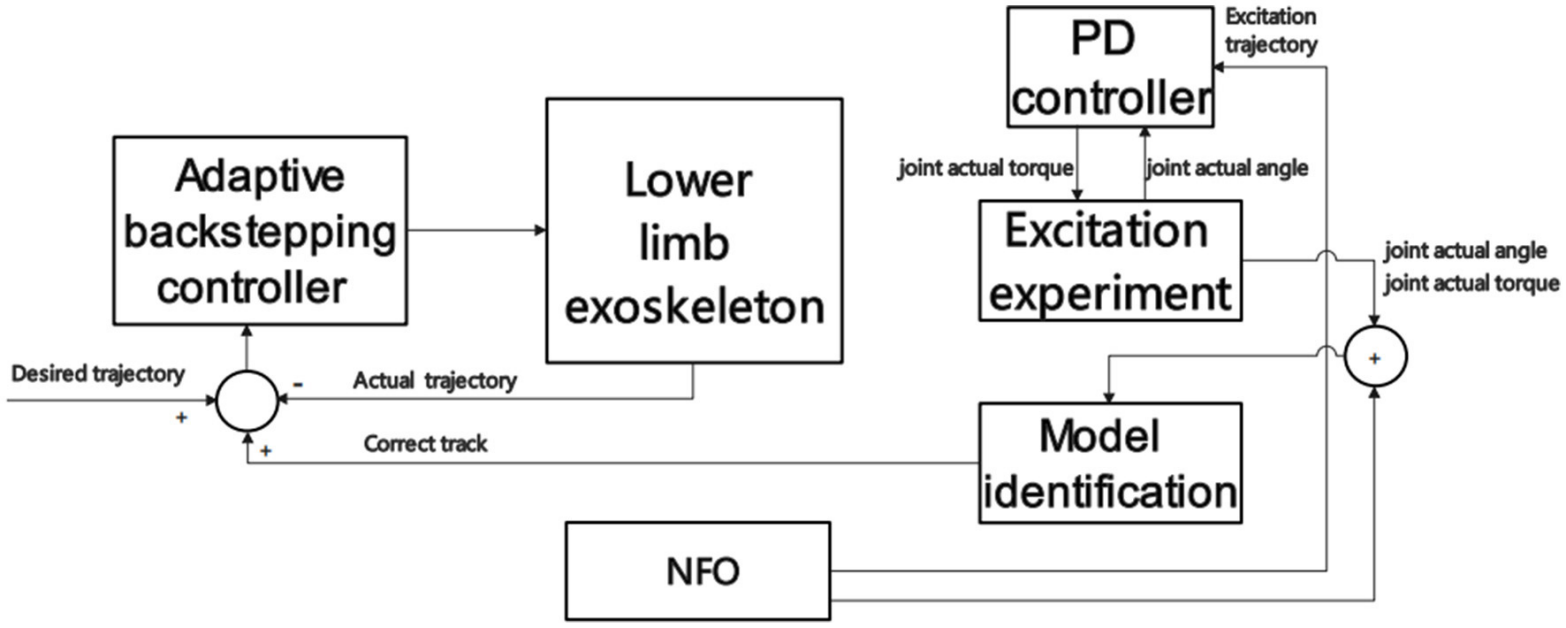
NFO-based control scheme.

Ding et al. (2018) collected kinematic parameters through an intelligent Inertial Measurement Unit (iIMU) and carried out movement intention recognition based on local search windows and fixed thresholds. This method responded quickly and realized the detection of human-machine synchronous walking gait events. However, the kinematic obtained by the IMU should be based on the characteristics of the inertia itself, and the range that can be predicted is limited. Chen et al. (2022) have made further advancements in intended recognition speed based kinematics. They have employed an AI algorithm known as Neighborhood Field Optimization (NFO) to address this challenge (Wu et al., 2012; Wu and Chow, 2013). Initially, they constructed the dynamic model of the exoskeleton using the Lagrangian modeling technique. Subsequently, they perform excitation experiments utilizing a Proportional-Derivative (PD) controller to acquire the kinematic parameters. These kinematic parameters are then used within the NFO framework to identify the model parameters. At this point, the desired trajectory of the exoskeleton corresponds to the actual motion trajectory, and the joint angles and velocities are incorporated into the feedback control mechanism. This approach significantly improves the precision and speed of intent recognition, enhancing adaptive control performance for the exoskeleton. NFO-based control scheme is shown in Figure 7.

Some studies have introduced plantar pressure to improve the accuracy of motion posture recognition. Sánchez Manchola et al. (2019) combined the IMU with the plantar pressure sensors to carry out movement intention recognition based on the collected signals. Comparing the training results found on the Threshold-based (TB) detection algorithm and Hidden Markov Model (HMM), the performance of the HMM-based is better, and it can realize the motion intention recognition of the gait cycle well and realize the adaptive control of exoskeleton. However, when this study was applied to stroke survivors, the effect of HMM was not satisfactory due to the weakness of the lower limbs of stroke survivors and the strong randomness in exercise. Moreover, using the IMU sensor, the joint vibration error generated will also affect the accuracy of motion intention recognition (Lee et al., 2014).

**Figure 8:**
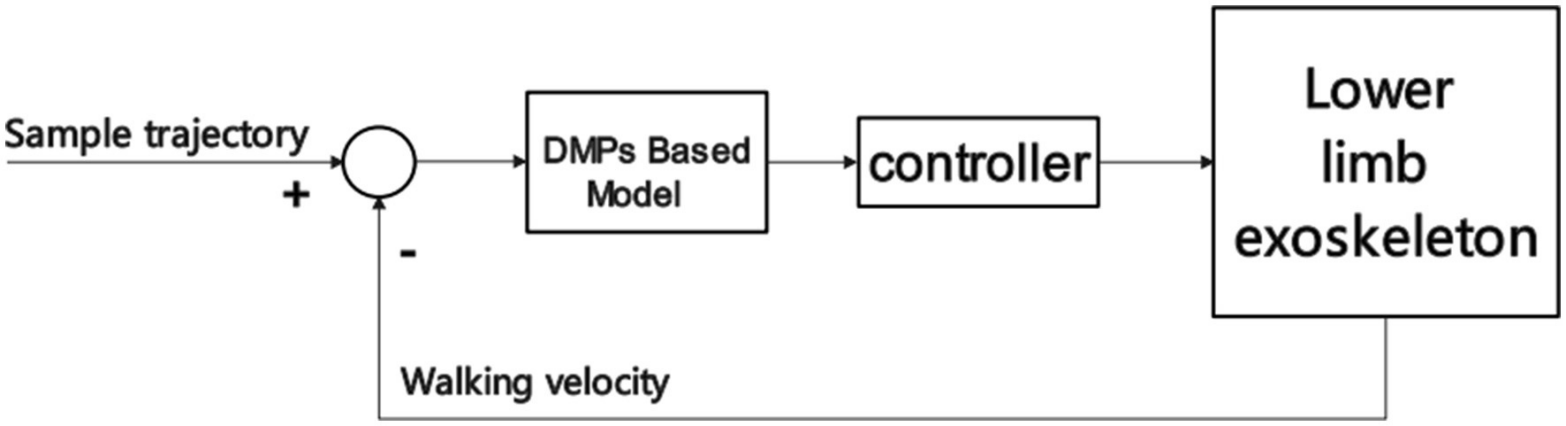
Scheme of DMPs-based control.

With the deepening of research in recent years, some scholars have also used optical, ultrasonic, and magnetic sensors to identify lower limb movement intentions (Roetenberg, 2006; Zhou and Hu, 2008; Maki et al., 2012). Among them, the optical method is the most widely used because, with the development of related research on NDI and Kinect in recent years, it has had a good effect on motion capture. Moreover, the somatosensory game developed by Microsoft's Xbox combined with Kinect for body recognition has been applied in the hospital's stroke rehabilitation training and has achieved specific results (Xavier-Rocha et al., 2020). More and more researchers have begun to try to recognize the lower limb movement intention based on the optical three-dimensional motion capture system. Ma et al. (2020) used VICON to measure different people's lower limb joint angle curves at different speeds, constructed a gait parameter model, and completed the exoskeleton adaptive control model through the gait prediction of the entire gait cycle planning through Dynamic movement primitives (DMP). The limitation of the optical sensor is that the detection angle of the sensor is fixed, the space range of the recognition is restricted, and stroke survivors need various safety protection during exercise, so the optical positioning mark is easily blocked, which affects the motion capture. These questions have led some scholars to use optical, IMU, and pressure sensors to collect relevant signals and realize adaptive control of lower limb rehabilitation exoskeletons based on multimodal information fusion and motion intention recognition as Section 4.3. Scheme of DMPs-based control is shown in Figure 8.

Nevertheless, the kinematic and kinetics can only be collected after the movement has occurred, and the signal's appearance lags behind the action's onset (small delay) (Pizzolato et al., 2017). Therefore, the model only estimates the following motion state and has limitations for adaptive control of the exoskeleton. To solve these problems, some scholars have also begun to try to realize motion intention recognition based on electrophysiological signals.

### 4.2. Adaptive model of lower limb exoskeleton for motion intention recognition based on electrophysiological signals

According to their acquisition methods, electrophysiological signals applied to motion intention recognition can be divided into invasive and non-invasive. The invasive acquisition method is to place electrodes into the human body. This acquisition method can obtain better signal quality, but it will cause trauma to the human body. The non-invasive acquisition method is to attach electrodes to the skin's surface. This acquisition method will not cause trauma to the human body, but the quality of the acquired signal is poor, and the signal needs to be pre-processed before the signal is used (Lew et al., 2012). The effective frequency of sEMG is 20–500 Hz. Considering the safety of signal acquisition and the physical condition of stroke survivors, lower limb Exoskeleton rehabilitation robots mostly use non-invasive sEMG for motion intention recognition. The sEMG is 10ms earlier than the muscle action, and the collected sEMG can be used for pattern recognition or deep learning to determine the movement intention (Huang et al., 2008; Ha et al., 2010; Hargrove et al., 2011; Wu et al., 2011; Hoover et al., 2012; Hardaker et al., 2013).

Liu et al. (2022) used a Metric-Learned Temporal Convolutional Network (ML-TCN) to extract and recognize sEMG features, thereby realizing gait phase recognition through sEMG. The method divides the gait cycle into 4 phases (heel strike, plantar strike, plantar lift, and foot swing). The study also compared Long Short-Term Memory (LSTM) models with those from standard Temporal Convolutional Networks (TCN). The experimental results of this study show that the temporal convolutional network test results of metric learning are better, and the recognition accuracy can reach 96.22%.

**Figure 9:**
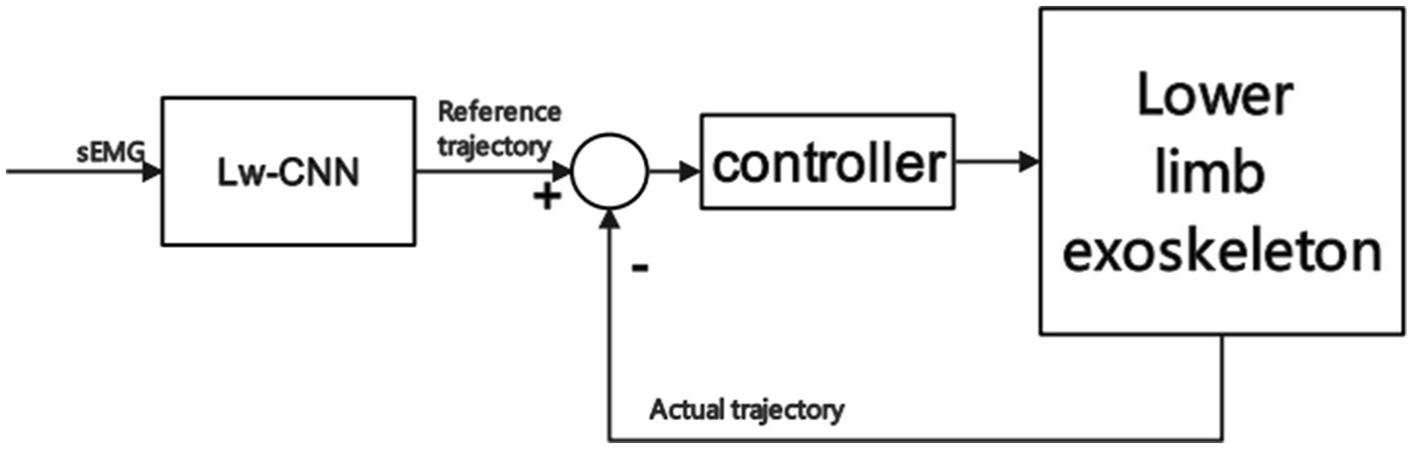
Scheme of adaptive control model based on Lw-CNN.

Since the motion intention recognition model applied to the lower limb exoskeleton has high requirements for real-time action, an overly complex model will lead to too long a recognition time, which cannot meet the real-time control of the exoskeleton. Therefore, Guo et al. (2020) uses a lightweight deep learning model, such as a light convolutional neural network (Lw-CNN), to classify and identify the upper arm sEMG that controls the robotic arm, and the robotic arm control accuracy can reach 88.75%. Scheme of adaptive control model based on Lw-CNN is shown in Figure 9.

However, long-term muscle exercise will cause muscle fatigue, which will cause the signal power spectrum to shift toward the low-frequency direction. The subsequent acquisition of sEMG will reduce the accuracy of model recognition and affect following intention recognition. For sEMG, it isn't easy to judge the starting point of the movement occurrence. The EEG has an advantage in recognition of action intentions, and the EEG is generated before the action occurs. Therefore, some scholars began to study EEG-based motion intention recognition.

For example, Choi and Kim (2019) performed feature extraction and classification through Mu-band event-related desynchronization (ERD) in EEG (Tariq et al., 2020). They also use the Modified Threshold Method for Time Series Classification (MTM-TSC) to reduce the classification error rate of gait detection and then realize the adaptive control of the exoskeleton. However, because the EEG signal is weak and susceptible to interference, and the steps of collecting EEG are cumbersome, stroke survivors must be highly concentrated during the collection. However, because the EEG signal is weak and susceptible to interference, the steps to collect EEG are cumbersome, and stroke survivors need to be highly concentrated during the collection process, which also limits the broad application of EEG. Subsequently, some scholars began to try to use a multi-source signal based on EEG and sEMG, integrate their advantages, and identify an adaptive control model through motion intention.

### 4.3. Adaptive model of lower limb exoskeleton for motion intention recognition based on multimodality

Today, lower limb exoskeleton rehabilitation robots tend to use various sensors, such as combined with IMU, plantar pressure, and others. At the same time, it can also collect sEMG and EEG and recognize the movement intention of lower limbs through multi-source signals. This improves the accuracy of motion intention recognition and can also use these sensors to do more work, such as rehabilitation assessment, fatigue monitoring, etc. (Ferrarello et al., 2013; Chang et al., 2017).

Hua et al. (2019) have developed an innovative Weight-Bearing Lower Limb Exoskeleton. They have integrated motor actuators to address the challenges of portability and flexibility under high loads in the exoskeleton. In addition, they have utilized stacked autoencoder deep neural networks (DNNs) based on multimodal signals to modify the exoskeleton's operation mode and control parameters. Remarkably, they have achieved an intent recognition accuracy of 99.7% through the utilization of a hybrid algorithm that combines genetic algorithm and particle swarm optimization (GA_PSO). Furthermore, they have successfully implemented the transition of gait cycle phases using an adaptive neural-fuzzy inference system (ANFIS). This innovative Weight-Bearing Lower Limb Exoskeleton demonstrates effective motion intent recognition and achieves adaptive control. Its control scheme is similar to common lower limb exoskeleton human-machine cooperative control as Figure 3.

Wang et al. (2022d) built an adaptive control model through spatial position information and motion mechanics parameters. The accuracy rate of motion intention recognition based on multi-source information reached 97.78%. The kinematic is convenient for obtaining parameters and does not affect the patient's rehabilitation training actions when collecting signals.

The kinematic provided by IMU and pressure-sensitive sensors cannot accurately identify spatial position information of stroke patients, so it is necessary to introduce motion capture to make compensation. Song et al. (2020), built a motion state recognition model based on feature evaluation and multi-layer BP (BackPropagation) neural network, collected kinematic, plantar pressure, and parameters collected by VICON, and performed multi-source feature parameter fusion. Finally, the two sets of multi-information source fusion models were verified, and the average recognition accuracy rate for 15 motion patterns was 95.05%. This study provided a good reference for the multi-source signal fusion intention recognition model in applying lower limb rehabilitation exoskeleton adaptive control example.

**Figure 10:**
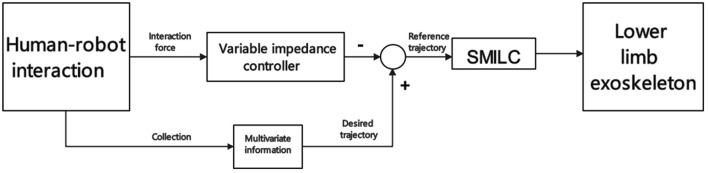
Dual-loop control system scheme.

To ensure real-time control of exoskeletons in stroke survivors, it is necessary to incorporate electrophysiological signals with lower latency than kinematic to recognize motion intentions. Du et al. (2018) have developed a variable impedance controller for lower limb exoskeletons based on sEMG and plantar pressure. Their objective is to optimize the reference motion trajectory of the exoskeleton. They use a sliding mode iterative learning controller (SMILC) based on the variable boundary saturation function, which enables accurate reference trajectory tracking and establishes a dual-loop control system. Notably, the proposed model mitigates undesired vibrations during the reference trajectory tracking process. Furthermore, it continuously adapts and optimizes the reference motion trajectory based on the recognition of motion intent. Dual-loop control system scheme is shown in Figure 10.

Likewise, Gui et al. (2019) obtained joint motion state by collecting sEMG and joint torque sensors' kinematic. Then, the radial basis function neural network (RBFNN) is used to train and identify the joint torque model, which can adaptively predict the joint motion state in the swing phase of the gait cycle in real time. And the study also showed that EEG signals generated by non-target muscles would interfere with the EEG of target muscles.

So Zheng et al. (2020) introduced EEG for intent recognition. They collected information such as EEG, joint position, and plantar pressure of the subjects, used Classical Correlation Analysis (CCA) (Liu et al., 2020) to identify the Steady-State Visual Evoked Potential (SSVEP) (Wang et al., 2010), combined with kinematic as the input of the machine learning model, constructed a multiple Modal machine learning model, which has an average accuracy rate of more than 90% in identifying SSVEP signals.

Some scholars also try to recognize motion intention based only on electrophysiological signals to realize adaptive control of exoskeletons in stroke survivors, but this method also has limitations (Net'uková et al., 2022). For example, to solve the problem of inaccurate recognition of subsequent sEMG acquisition due to muscle fatigue during long-term exercise, Al-Quraishi et al. (2021), Discriminant Correlation Analysis (DCA)-Based Modal Fusion of EEG and sEMG to identify lower limbs Movements.

Although motion intention recognition based on EEG and sEMG multi-source information can effectively improve the control precision of the model and the accuracy of intention recognition, the response time of different physiological signal sources is different, and there is an unavoidable delay (Zhang et al., 2021). This model has a specific difficulty in the construction of the model. This problem is also the main challenge of multi-source signal fusion at present. For example, the response of kinematic collected by IMU occurs later than that of electrophysiological signals, and additional processing is required to match and fuse them (Net'uková et al., 2022). Second, the adaptive control algorithm for motion intention recognition based on multi-source information requires a longer computation time than the general model. This model also impacts the real-time application of the adaptive control model on the lower limb exoskeleton rehabilitation robot; this problem also affects the real-time application of the adaptive control model on the lower limb exoskeleton rehabilitation robot (Zhang et al., 2022).

**Figure 11:**
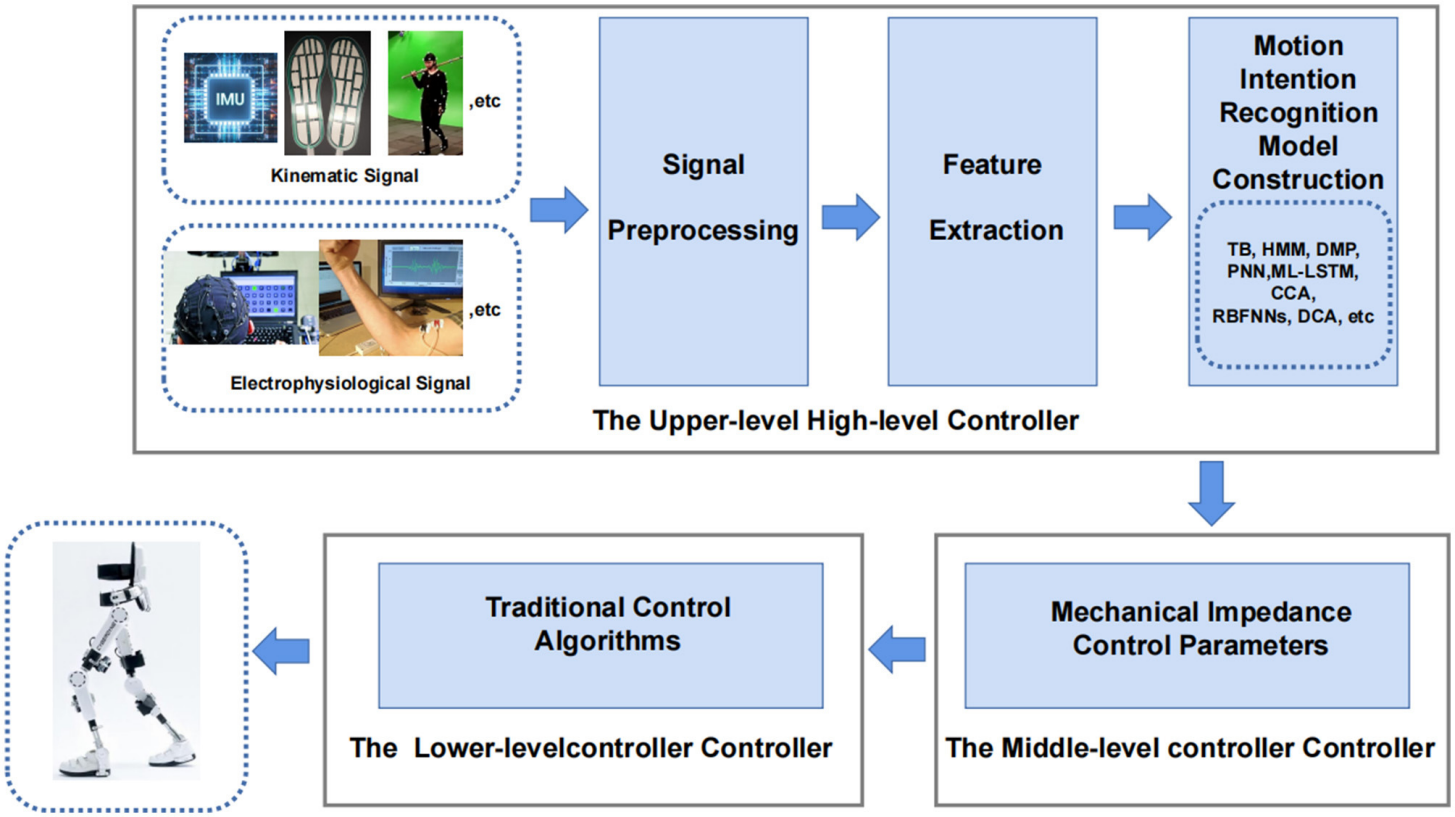
Hierarchical architecture of lower limb rehabilitation exoskeleton.

Most adaptive control exoskeletons based on motion intention recognition now use a layered architecture to achieve control. The upper-level high-level controller is used for motion intention recognition, and the middle-level controller converts the motion pattern predicted by intention recognition into mechanical impedance control parameters. The lower-level controller uses traditional control algorithms to drive actuators to achieve closed-loop control are shown in Figure 11.

## 5. Related review comparison

This section collects the review of lower extremity exoskeletons in the past three years, and their main work content is summarized and compared.

Gholap et al. (2022) is a review of exoskeleton system development. In the review, the biomechanical parameters of the human body are also discussed to understand the various degrees of freedom of the human body's joints and to recognize their significance in the gait cycle. In addition, different existing exoskeletons and actuators used in exoskeletons are analyzed comparatively.

The work of de Miguel-Fernández et al. (2023) is to evaluate the rehabilitation effect of different control algorithms for the lower extremity exoskeleton for gait rehabilitation after brain injury. Includes: providing an updated structured framework of current control strategies, analyzing clinically validated methods used in robotic interventions, and reporting potential relationships between control strategies and clinical outcomes. They find only 14% of the exoskeletons were found to implement adaptive control strategies.

Masengo et al. (2023) discusses exoskeleton control methods, including physiological-based control (sEMG and EEG) and traditional-based cooperative control of lower extremity exoskeleton control, which are reviewed and compared in depth. And discuss the trend of multi-source fusion.

Jiyu et al. (2022) review focus on the portability, intelligent control, and modular structure design of the lower extremity exoskeleton. It pays more attention to the materials used, modular design, human-computer interaction interface, cloud data, security, and intelligence of the exoskeleton, energy efficiency, and cost-effectiveness.

Lee et al. (2020) review focus on three classes of lower extremity exoskeletons: assistive, rehabilitation, and augmented exoskeletons. This review is introduced around its control actuator type.

Wang et al. (2022c) the review focuses on the overall design of the exoskeleton, drive unit, intent perception, compliance control, and efficiency verification. Also, it discusses the complexity and coupling of the human-machine integrated system.

Al-Rahmani et al. (2022) summarize the design, working principles, and applications of robotic devices for foot drop assistance and rehabilitation over the past decade. The findings describe the design aspects of 72 lower limb robotic assistive devices, including 21 studies evaluating specific design aspects through experimental trials, and discuss the future potential of advanced robotic devices for foot drop assistance and rehabilitation.

We can see that the current review research questions of lower limb exoskeletons mainly focus on the following aspects.

### 5.1. Safety of lower limb exoskeleton rehabilitation robot

The lower limb rehabilitation exoskeleton robots need human-machine coupling to assist or strengthen humans to complete required actions (Lee et al., 2020). Therefore, when using, the user's safety should be put first, and avoid causing trauma due to an unstable control system or unreasonable mechanical structure design during use. The control system of exoskeleton robots applied to stroke survivors should be more stable and able to brake in time. Stroke survivors have movement disorders, and coping with sudden lower limb exoskeleton emergencies is even more challenging. Therefore, safety indicators must be considered when designing lower limb exoskeletons. For example, set the maximum speed of movement, the upper limit of the maximum torque of the actuator, etc. In addition, the lower limb exoskeleton needs to provide adaptive weight support according to the patient's condition and muscle strength, which can prevent stroke survivors from falling during exercise and can also adaptively adjust the exercise load of stroke survivors. Wu et al. (2021), did related research on this work and first eliminated random disturbances through the linear quadratic exponential optimal control method. Estimable system disturbances introduced by the user due to gravity changes are then eliminated by a feed-forward control method based on error inputs. Finally, the Body Suspension Model (BSM) model was established, and the exoskeleton adaptively provided weight support was successfully realized.

### 5.2. Objective rehabilitation assessment based on multimodal information fusion

Traditional approaches to rehabilitation assessment have been enumerated previously. As introduced in Section 2, the rehabilitation assessment method combined with the rehabilitation scale is still widely used in hospitals. It has crucial diagnostic significance, but the instrumental assessment has gradually become the new gold standard for rehabilitation assessment (Toro et al., 2003; Rathinam et al., 2014). First, the lower limb rehabilitation exoskeleton robots can help physicians assist patients in rehabilitation training. In addition, as mentioned before, it is feasible to combine various sensors and electrophysiological parameters to evaluate the current rehabilitation of patients, which is consistent with the movement mechanics and electrophysiological parameters required for motion intention recognition and will not increase high additional research costs.

Therefore, in the future, the lower limb rehabilitation exoskeleton robot based on motion intention recognition can integrate multi-source information and construct a multimodal rehabilitation evaluation model through machine learning or other technologies. Realize an objective rehabilitation assessment method based on multimodal information fusion. This can enable stroke survivors to complete rehabilitation assessments during rehabilitation training and save the assessment results. Rehabilitation therapists can directly modify the follow-up rehabilitation plan based on the evaluation opinions given by the lower limb rehabilitation exoskeleton robot, which reduces the workload of rehabilitation therapists and allows rehabilitation physicians to perform more valuable diagnostic work.

### 5.3. Coping with complex tasks or situations

Most adaptive lower limb rehabilitation exoskeleton robots based on motion intention recognition are currently researched on simple gait cycle motions, such as walking on flat ground or a flatbed. More research still needs to be done on the movement of various complex scenes. Winter et al.'s (2021) research shows that stroke survivors can effectively increase exercise motivation and improve therapeutic effects when using virtual reality to imitate the outdoor environment for immersive training. This provides us with a new way of thinking, which can extend the application scenarios of the adaptive lower limb rehabilitation exoskeleton robot based on motion intention recognition to the outdoors. Alternatively, apply the virtual reality method to simulate the outdoor environment and simultaneously increase the training obstacles to simulate various problems encountered in the natural environment.

For this reason, the adaptive lower limb rehabilitation exoskeleton needs to cope with complex scenes and perform some complex movements, such as going up and down stairs. Laschowski et al. (2020), combined laser rangefinder and radar detector, built an exoskeleton environment recognition system through image classification recognition, machine learning, and convolutional neural network, and successfully recognized complex environments. Zhong et al. (2021) took this research a step further. Collect environmental information through self-made embedded hardware, and use IMU sensor for motion intention recognition. Furthermore, the BNN (Bayesian neural network, BNN) is used to predict the collected information parameters, and the classification of six complex terrains is completed. This model can also be extended to multi-modal fusion, and motion intention recognition based on multi-modal fusion can realize adaptive control of the complex environment of the lower limb rehabilitation exoskeleton. The adaptive lower limb rehabilitation exoskeleton robot based on motion intention recognition integrates the environment recognition function and can achieve more complex tasks. In the follow-up research, related work can be continued to make the application scenarios of the lower limb rehabilitation exoskeleton more diverse, which can help stroke survivors perform rehabilitation training in different scenarios, strengthen the efficacy of training, and perform more complex rehabilitation training tasks.

### 5.4. Lower limb exoskeleton cable or non-cable

Currently, there are two common ways of the lower limb rehabilitation exoskeleton: wired and wireless. Through a wired connection, data transmission is more stable. The disadvantage is that too many wires will interfere with the subject's movement to a certain extent and limit the subject's range of activities, And there are certain requirements on the ratio of cable length (Prasad et al., 2022; Zuccon et al., 2022). The wireless connection method is more portable and constitutes a wireless body area network. Generally, it will not interfere with the activities of the subjects, but in exceptional cases, the transmission of data may be interfered with, resulting in inaccurate identification of intentions. Since stroke survivors are not very athletic, wired sensor data transmission can be used in combination with virtual reality technology to simulate outdoor training to ensure the stability of data and the accuracy of intention recognition. In addition, the adaptive control model of multi-modal fusion also increases the computational pressure of the model to a certain extent, so it is also very critical to choose an appropriate algorithm to reduce the delay of the exoskeleton response.

### 5.5. Combining lower limb exoskeleton rehabilitation robots with other stroke treatments

When stroke survivors undergo rehabilitation training, if they rely entirely on exoskeleton rehabilitation robots for support, exercise will lead to less active participation in their movement. At this time, the muscles of the lower limbs and the knees receive less reaction from the ground and are not significantly stimulated, so the therapeutic effect is not apparent. The adaptive control exoskeleton robot using electrophysiological signals for motion intention recognition has more advantages in stimulating nerves and muscles than the adaptive control exoskeleton robot using sensors for motion intention recognition. Because the collected electrophysiological signals are mainly generated by motor imagery or muscle movement of stroke survivors, it is beneficial to the brain function remodeling of stroke survivors.

To enhance the intensity of stimulation to the muscles and nerves of stroke survivors, the lower limb rehabilitation exoskeleton robot can be combined with other stroke treatment methods. For example, by combining functional electrical stimulation with the lower limb rehabilitation exoskeleton robot, through the adaptive control strategy of the exoskeleton based on functional electrical stimulation, the change of the joint angle of the subject is recognized to adjust the electrical stimulation current intensity and change the joint motion state. This not only realizes the adaptive control of the exoskeleton but also corrects the user's gait and simultaneously enhances the stimulation of the lower limb muscles to achieve better rehabilitation effects. Therefore, in the future, when designing exoskeleton-related control methods, other methods of stroke rehabilitation can be considered.

## 6. Conclusions

The main contribution of this review is the introduction of today's instrumented rehabilitation assessment methods, emphasizing the necessity of adaptively controlled lower limb exoskeleton robots with rehabilitation assessment functions, and the study also introduces the lower limb exoskeleton based on various actuators. The exoskeleton robot control model for lower limb rehabilitation based on motion intention recognition based on multiple AI methods is mainly reviewed.

In the future, in designing lower limb rehabilitation exoskeleton robots for stroke survivors, it is necessary to focus on the safety of the exoskeleton and adapt the weight reduction system to cope with stroke survivors of different muscle strength levels. At the same time, to meet the application of stroke survivors throughout the rehabilitation stage, the lower limb exoskeleton robot needs to have the function of rehabilitation assessment. To achieve a better rehabilitation effect, the training content that the exoskeleton can perform should be expanded, and some complex tasks can be performed or combined with virtual reality technology. The adaptive control model based on motion intention recognition can extract the motion intention of stroke survivors, adjust exoskeleton parameters in real-time, control exoskeleton movement, and promote brain function remodeling in stroke survivors. The multi-modal motion intention recognition can make the predicted results more accurate, so future research should focus on the multi-modal motion intention recognition adaptive control model research. In addition, the lower limb rehabilitation exoskeleton robot has a limited role in the rehabilitation treatment process, so the limb rehabilitation exoskeleton robot can be combined with other stroke rehabilitation treatment methods to achieve better efficacy. Overall, there is still much room for developing exoskeleton robots for lower limb rehabilitation for stroke survivors.

## Author contributions

DS and ZL organized the graphs and tables. DS wrote the first draft of the manuscript. PS, JW, ZL, and ZH wrote sections of the manuscript. All authors contributed to manuscript revision, read, and approved the submitted version.
